# Design and Analysis of Area and Energy Efficient Reconfigurable Cryptographic Accelerator for Securing IoT Devices

**DOI:** 10.3390/s22239160

**Published:** 2022-11-25

**Authors:** Xvpeng Zhang, Bingqiang Liu, Yaqi Zhao, Xiaoyu Hu, Zixuan Shen, Zhaoxia Zheng, Zhenglin Liu, Kwen-Siong Chong, Guoyi Yu, Chao Wang, Xuecheng Zou

**Affiliations:** 1 School of Optical and Electronic Information, Huazhong University of Science and Technology, Wuhan 430074, China; 2 Wuhan National Laboratory of Optoelectronics, Huazhong University of Science and Technology, Wuhan 430074, China; 3 Temasek Laboratories, Nanyang Technological University, Singapore 639798, Singapore; 4 Zero-Error Systems Pte Ltd., TCH Techcentre, 71 Toh Guan Road East, #05-07, Singapore 608598, Singapore

**Keywords:** reconfigurable cryptographic accelerator, hardware security, intelligent sensors and mobile robots, intelligent Internet of Things

## Abstract

Achieving low-cost and high-performance network security communication is necessary for Internet of Things (IoT) devices, including intelligent sensors and mobile robots. Designing hardware accelerators to accelerate multiple computationally intensive cryptographic primitives in various network security protocols is challenging. Different from existing unified reconfigurable cryptographic accelerators with relatively low efficiency and high latency, this paper presents design and analysis of a reconfigurable cryptographic accelerator consisting of a reconfigurable cipher unit and a reconfigurable hash unit to support widely used cryptographic algorithms for IoT Devices, which require block ciphers and hash functions simultaneously. Based on a detailed and comprehensive algorithmic analysis of both the block ciphers and hash functions in terms of basic algorithm structures and common cryptographic operators, the proposed reconfigurable cryptographic accelerator is designed by reusing key register files and operators to build unified data paths. Both the reconfigurable cipher unit and the reconfigurable hash unit contain a unified data path to implement Data Encryption Standard (DES)/Advanced Encryption Standard (AES)/ShangMi 4 (SM4) and Secure Hash Algorithm-1 (SHA-1)/SHA-256/SM3 algorithms, respectively. A reconfigurable S-Box for AES and SM4 is designed based on the composite field Galois field (GF) GF(((2^2^)^2^)^2^), which significantly reduces hardware overhead and power consumption compared with the conventional implementation by look-up tables. The experimental results based on 65-nm application-specific integrated circuit (ASIC) implementation show that the achieved energy efficiency and area efficiency of the proposed design is 441 Gbps/W and 37.55 Gbps/mm^2^, respectively, which is suitable for IoT devices with limited battery and form factor. The result of delay analysis also shows that the number of delay cycles of our design can be reduced by 83% compared with the state-of-the-art design, which shows that the proposed design is more suitable for applications including 5G/Wi-Fi/ZigBee/Ethernet network standards to accelerate block ciphers and hash functions simultaneously.

## 1. Introduction

Internet of Things (IoT) devices, including intelligent sensors and mobile robots, often collect information from the environment, and transmit a large amount of data to the cloud and other IoT devices. This large amount of data needs to be managed, processed, transferred and stored securely [1,2]. To enable IoT end-to-end security, cryptographic primitives such as block ciphers and/or hash functions in the IoT devices need to support multiple network communication standards (such as 5G, Wi-Fi, ZigBee, and Ethernet [3]), to ensure communication privacy and integrity [4], and to abide by various security protocols regulated by various security standards and countries. The block ciphers include Data Encryption Standard (DES), Advanced Encryption Standard (AES) and ShangMi 4(SM4), whereas the hash functions include Secure Hash Algorithm-1 (SHA-1), SHA-256 and SM3 [5,6]. Although these various cryptographic primitives can easily be implemented at the software level [7] (e.g., by using a microcontroller), unfortunately the software implementation often results in high latency and large energy consumption overheads, hence limiting deployment on IoT devices which are highly resource constrained.

To mitigate the high latency and large energy consumption overheads, cryptographic accelerators for various cryptographic primitives are often adopted. The common practice includes the realization of a dedicated cryptographic accelerator for one specific cryptographic primitive. However, such a practice cannot support multiple cryptographic primitives as required by the emerging security protocols for IoT devices [8,9]. The direct implementation of each cryptographic primitive, with its dedicated cryptographic accelerator, causes large hardware overhead and energy consumption, which is similarly not an efficient solution for resource-constrained IoT devices. Alternatively, the idea of configuring cryptographic accelerators to support multiple cryptographic primitives has been reported. Liu et al. [10] have reported a dynamically reconfigurable processing array for supporting DES/AES/SM4 and SHA-256/SM3. Du et al. [11] have reported a coarse-grained reconfigurable cryptographic logic array and an intelligent mapping algorithm for different cryptographic algorithms, such as DES/AES/SM4/SHA-256, etc. Deng et al. [12] have reported a coarse-grained reconfigurable cryptographic processor, which can implement all the commonly used cryptographic algorithms, including DES, AES, SM4, SHA-256, SM3, etc.

These reported configurable cryptographic accelerators, however, remain inefficient for three reasons. First, they adopt a unified hardware unit approach by integrating both block ciphers and hash functions into a common unit, and are hence unable to process both block cipher and hash functions simultaneously. Considering some actual application scenarios, the protocols, including the Wi-Fi Protected Access (WPA) widely used in Wi-Fi, and the Datagram Transport Layer Security (DTLS)/Internet Protocol Security (IPsec) protocol widely used in ethernet standards [7,9,10], require both encryption and hash functions to be performed simultaneously. The series processing of the unified hardware unit increases latency. Second, the unified hardware unit is not circuit optimized because it has to accommodate distinct differences in terms of data paths and logic operations between the block ciphers and hash functions. Third, they adopt look-up tables (LUTs) to implement the substitution box (S-Box), which causes large hardware overhead and is therefore not an efficient solution.

In this paper, a reconfigurable cryptographic accelerator is proposed that has three-tier configurability (i.e., functional configurability, data-path configurability, and S-Box configurability) to support widely used security functions for resource-limited and energy-constrained IoT devices including small mobile intelligent robots, wearable medical devices and miniature unmanned aerial vehicles. The major contributions of the paper are as allows.

1. At tier1 of the functional configurability, the proposed reconfigurable cryptographic accelerator can perform either block ciphers (DES/AES/SM4) alone, or hash functions (SHA-1/SHA-256/SM3) alone or both block cipher and hash function simultaneously. This is achieved by having two separate unified reconfigurable engines (i.e., one for the block ciphers, and another for the hash functions), that account for latency optimization for actual application scenarios including authentication and encryption/decryption processes.
2. At tier2 of the datapath configurability, the datapath for the block ciphers and for the hash functions are optimized. This is achieved by first having a detailed and comprehensive algorithmic analysis of both the block ciphers and hash functions in terms of basic algorithm structures and common cryptographic operators to guide efficient reconfigurable architecture design, which provides a practical design reference for the reconfigurable hardware design of cryptographic algorithms. Through this analysis, in the proposed design implementation, the datapath for the block ciphers is optimized to have a 33.65% smaller area, and the datapath for the hash functions a 56.18% smaller area.
3. At tier3 of the S-Box configurability, a reconfigurable S-Box module for AES and SM4 is designed based on the composite Galois field (GF) GF(((2^2^)^2^)^2^) instead of GF(2^8^), which significantly reduces hardware overhead by more than 30% as compared to the conventional implementations of LUTs.

The remainder of this paper is organized as follows: Section 2 provides an algorithm analysis of both block ciphers and hash functions. Section 3 describes the architecture and hardware implementation of the proposed reconfigurable cryptographic accelerator. Section 4 presents experimental results and discussions. Section 5 presents the conclusion.

## 2. Algorithm Analysis of Block Ciphers and Hash Functions

This section first provides a brief review of the application scenarios of block ciphers and hash functions and then presents an introduction to the structures and principles of the cryptographic algorithms.

### 2.1. Review of Cryptographic Algorithms in IoT Security Applications

Edge IoT devices face the security threat of data disclosure during communication. Various IoT network standards that apply different cryptographic algorithms are an important means to ensure communication security. For communication security, ensuring the privacy and integrity of transmitted data is a basic requirement. Block ciphers and hash functions are two categories of major cryptographic algorithms that have been widely used in various IoT network standards to ensure data privacy and integrity, respectively. Block cipher is a symmetric cryptographic algorithm that performs encryption or decryption on a fixed-size data block using a shared secret key. Plaintext is the source data during the encryption, and the resulting encrypted text is called ciphertext. The same key is used for both the encryption of plaintext and the decryption of ciphertext. Hash function is a type of security mechanism that produces a hash value, message digest or checksum value for a specific data object. If the data are intentionally or unintentionally modified, the hash value is changed. Thus, the integrity of data objects can be evaluated by comparing and verifying previous and current hash values of the transmitted data.

Table 1 shows major algorithms of block ciphers and hash functions that have been widely used in different security protocols and communication standards. It can be found that DES/AES/SM4 and SHA-1/SHA-256/SM3 are the two major algorithm groups for block ciphers and hash functions, respectively, which are widely deployed in major countries/regions for various network application scenarios. Notably, some of the key cryptographic algorithms need to be used at the same time. For example, DES/AES/SM4 and SHA-1/SHA-256/SM3 are simultaneously used to perform encryption and authentication, respectively, as part of the Internet Protocol Security (IPsec), which is a set of protocols widely used for the secure establishment of virtual private networks (VPNs). Similarly, AES/DES/SM4 and SHA-1/SHA-256/SM3 are also simultaneously used in the Transport Layer Security (TLS), which is a security protocol used to secure data transmitted over a network by preventing data from being eavesdropped on or tampered with. These security protocols, using different cryptographic algorithms, are able to support various communication standards, including 5G, Wi-Fi, ZigBee, and Ethernet, which are the key to achieving seamless network communication among the IoT devices in the different networks deployed in different countries and regions. Therefore, DES/AES/SM4 and SHA-1/SHA-256/SM3 algorithms were selected in this study for reconfigurable hardware accelerator design, which is able to effectively support IoT security for major countries and regionstitute of Standards and Technology.

### 2.2. Introduction of Major Block Ciphers and Hash Functions

#### 2.2.1. Algorithm Analysis of Major Block Ciphers

Block ciphers usually utilize multiple iterations of round operations on data blocks to implement confusion and diffusion functions that ensure strong data security. There are two major constructions of round operations in block ciphers: Feistel networks and Substitution–Permutation (SP) network [13,14].

**Figure 1:**
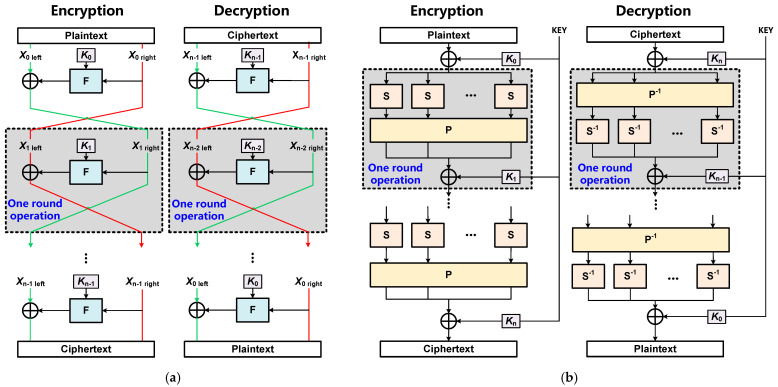
(**a**) Round operation structure of Feistel, (**b**) Round operation structure of SP.

Figure 1a presents a Feistel network containing n same-round operations. Each round operation contains a key-schedule operation generating round key *K*_i_ (i ∈ {1, 2, ···, n}), a round function F and a consequent XOR operation. The inputs of the round function F are data block *X*_i right_ from the former round operation (excluding the initial plaintext input *X*_0 right_ of the first-round operation) and the round key *K*_i_. The round function F contains basic operations including addition, S-Box, shift, and permutation to realize the confusion and diffusion of plaintext data, which have been adopted in the DES/SM4 algorithms. The purpose of confusion is to make the statistical relationship between plaintext and ciphertext as complex as possible, while diffusion is used to quickly spread the statistical characteristics of the plaintext into the ciphertext. The output of the round function F is XORed with the remaining data block *X*_i left_ from the former round operation (excluding the initial plaintext input *X*_0 left_ of the first-round operation). After n same-round operations, the final output is the ciphertext. It is worth noting that the Feistel network-based encryption and decryption have the same operation structure, and the only difference is that the round keys are applied in the reverse order in the decryption process as shown in Figure 1a.

Figure 1b shows the construction of an SP network also containing n same round operations. Each round operation applies several substitution boxes (S-Boxes), a permutation box (P-Box) and a consequent XOR operation on the plaintext block to produce the ciphertext block, which has been used in the AES algorithms. The input of the S-Boxes in each round operation is from the former round operation (excluding the first-round operation taking the XOR of plaintext and the initial round key *K*_0_ as input). The S-Boxes and P-box can efficiently realize confusion and diffusion of plaintext data, respectively. The S-Boxes realize the nonlinear permutation of block data, while a P-box realizes the permutation of all the data bits, which takes the outputs of all former S-Boxes in the same-round operation and permutes the data bits. The output of the P-box is XORed with the round key *K*_i_ generated by the key-schedule operation to complete one round operation. Similarly, after n same-round operations, the final output is the ciphertext. It is worth noting that the decryption of the SP-based algorithm is the inverse operation of the encryption process as shown in Figure 1b.

**Figure 2:**
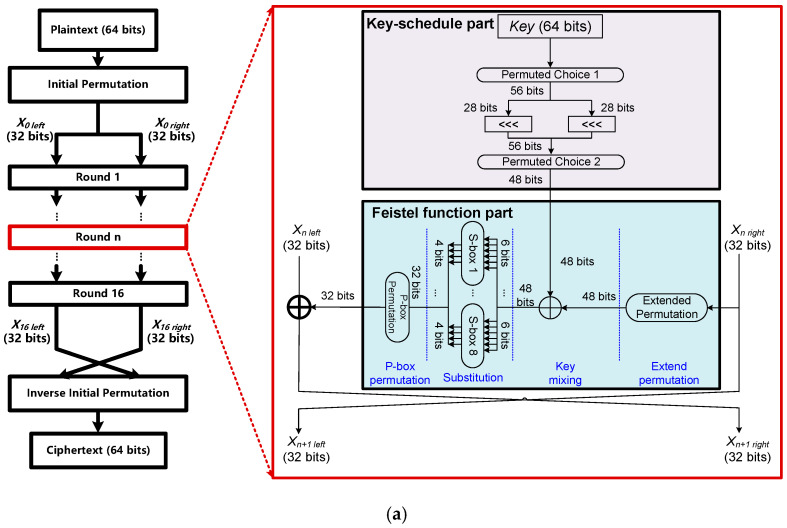

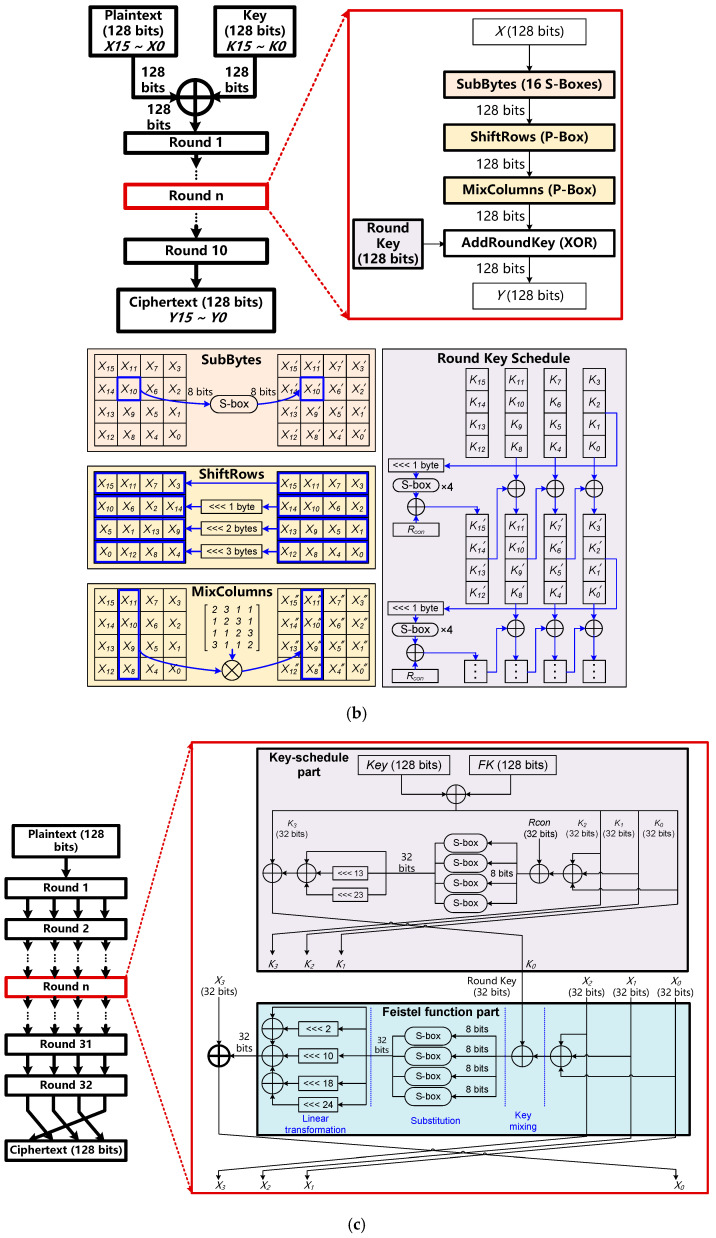
(**a**) The algorithm flowchart of DES based on the Feistel network, (**b**) The algorithm flowchart of AES based on the SP network, and (**c**) The algorithm flowchart of SM4 based on the Feistel network.

Figure 2a shows the data flow of the DES algorithm with a 64-bit input block, a 64-bit key size and a 64-bit output block. For the encryption process, there is an initial permutation, 16 round operations and a final permutation. After the initial permutation, the 64-bit data block is divided into two 32-bit halves (*X*_0 left_ and *X*_0 right_) and subsequently processed by the Feistel round operators in a crisscrossing manner. There are two major parts in each round operation, i.e., the key-schedule part and the Feistel function part, as shown in Figure 2a. For the key-schedule part, the 56 bits of the key are initially selected from the initial 64 bits by permuted choice 1, divided into two 28-bit halves, and then rotated left by one or two bits (specified for each round) in successive rounds. Next, 48-bit round key are selected from the 56-bit input by permuted choice 2 with 24 bits from the left, and 24 bits from the right. For the Feistel function part, there are four steps, including the extended permutation, key mixing, substitution and P-box permutation. In the extended permutation, the 32-bit right half-block (*X*_n right_) is expanded to 48 bits using the expansion permutation. In the key mixing, the 48 bits from extended permutation are XORed with the key in this round. In the substitution, the 48-bit block is divided into eight 6-bit blocks and eight S-Boxes are used to replace the 6-bit input with 4-bit output according to a non-linear transformation. In the P-box permutation, the eight 4-bit outputs from the eight S-Boxes are rearranged according to a P-box permutation to achieve diffusion as mentioned before. The 32-bit output of the Feistel function part is XORed with the 32-bit left half-block (*X*_n left_) to get the final result of one round operation. For the decryption process, the only difference from the encryption process is that the round keys are applied in reverse order in the decryption process.

Figure 2b shows the data flow of the AES-128 algorithm with a 128-bit input block, a 128-bit key and a 128-bit output block as an example to illustrate the AES algorithms with 128/192/256-bit keys. As compared to the AES-128 algorithm with 10-round operations, the AES-192 and AES-256 algorithms also process on a block of 128-bit data but with 192-bit and 256-bit keys, respectively, which corresponds to 12 and 14 rounds of operations, respectively. Different from the DES algorithm, the AES uses the SP structure instead of the Feistel structure. For the encryption process, there are four major steps, i.e., SubBytes, ShiftRows, MixColums and AddRoundKey, in one round operation. In the SubBytes step, each byte *X*_i_ (i = 0–15) is permuted using an 8-bit S-Box and there are a total 16 S-Boxes used in this step. In the ShiftRows step, data bytes in each row are shifted to the left, cyclically excluding the first row. In the MixColums step, each column is multiplied with a fixed matrix. In the AddRoundKey step, each byte is XORed with a byte of the round key. For the round key schedule process, the right column (*K*_0_, *K*_1_, *K*_2_, *K*_3_) is first cyclically shifted to the left with one byte. Then four S-Boxes are used to perform byte substitution. Next, the results from the four S-Boxes and *R*_con_ are XORed with the left column (*K*_12_*, K*_13_*, K*_14_*, K*_15_) to get a new column (*K*_12_^′^, *K*_13_^′^, *K*_14_^′^, *K*_15_^′^). Finally, the remaining three columns of round keys can be obtained as shown in Figure 2b. It is to be noted that the last round operation does not include the MixColumns step. For the decryption process, InvShiftRows (the inverse of ShiftRows), InvSubBytes (the inverse of SubBytes), AddRoundKey (XOR operation using the inverse order of round key) and InvMixColums (the inverse of MixColums) are used in each round operation.

Figure 2c shows the data flow of the SM4 algorithm with a 128-bit input block, a 128-bit key and a 128-bit output block, which is the same as the AES-128. There are 32 round operations to calculate the final ciphertext. Similar to the DES, the SM4 also uses the Feistel structure in each round operation and there are also a key-schedule and a Feistel function part. For the key-schedule part, the 128-bit original key is XORed with fixed parameter FK, and then the result is divided into four 32-bit data, i.e., *K*_0_, *K*_1_, *K*_2_ and *K*_3_. The *K*_0_, *K*_1_ and *K*_2_ are XORed with a 32-bit fixed parameter *R*_con_, and then the result is divided into four 8-bit data, which are input to four S-Boxes. The four results from four S-Boxes are combined into one 32-bit datum, which is shifted left cyclically by 13 and 23 bits. The original and shifted data are XORed with *K*_3_ to get *K*_0_ in the next round as shown in Figure 2c. Similar to the key-schedule part, for the Feistel function part, the 128-bit block is divided into four 32-bit data, i.e., *X*_0_, *X*_1_, *X*_2_ and *X*_3_. The *X*_0_, *X*_1_ and *X*_2_ are first XORed with a 32-bit round key *K*_0_ to get a 32-bit result, which is divided into four 8-bit data and input into four S-Boxes to perform the substitution. The four results from S-Boxes are combined into a 32-bit output data, which will be shifted left cyclically by 2, 10, 18 and 24 bits respectively to obtain four 32-bit data. The four data are XORed with the original data to get one result to complete the Feistel operation. Finally, *X*_3_ is XORed with the 32-bit Feistel result to get *X*_0_ in the next round as shown in Figure 2c. For the decryption process, similar to DES, the only difference from the encryption process is that the round keys are applied in the reverse order in the decryption process.

Table 2 summarizes the structure types and key operators of round operations in the three major block ciphers. It can be seen that both the DES and SM4 algorithms use the same round operation structure, i.e., Feistel structure, which means that the encryption and decryption data paths of DES/SM4 can be designed to a unified hardware. However, we can also find that, in fact there are more similar basic operators in the round operation of AES and SM4 algorithms, which can be exploited in order to reuse operators in the reconfigurable design to reduce hardware overhead.

Table 3 shows detailed information on key operators of round operations in the three major block ciphers. It can be seen that the key basic operators of SM4 are also included in the AES, while the majority of key basic operators in DES are different from the AES and SM4. It is worth noting that the same operators in different algorithms can be theoretically reused in hardware implementation. However, reusing operators also requires additional control hardware overhead, which reduces or even counteracts the advantage of reduced hardware overhead by reusing operators, especially for some simple logic operators or logic operators that are used less frequently. The 8-8 bit S-Box has the largest hardware overhead and the most times of usage, which is a key operator that needs to be reused. It is to be noted that for the AES and SM4 algorithms, because the mapping relationship between input and output of S-Box is inconsistent, there is no reusable part of the S-Box that is directly implemented based on look-up table. However, in the implementation of the S-Box based on Galois field, the calculation process of the two algorithms has the same inverse operation under GF (2^8^) that will be introduced in detail in Section III, which can be exploited for reconfigurable design. In addition to the operators, reusing a large number of data iteration register files frequently used in round operations is also the key to building a unified data path.

From the above analysis, the key idea of the reconfigurable hardware design of block ciphers is to build a unified cipher data path by reusing the reconfigurable S-Boxes based on Galois field and data iteration register files.

#### 2.2.2. Algorithm Analysis of Major Hash Functions

**Figure 3:**
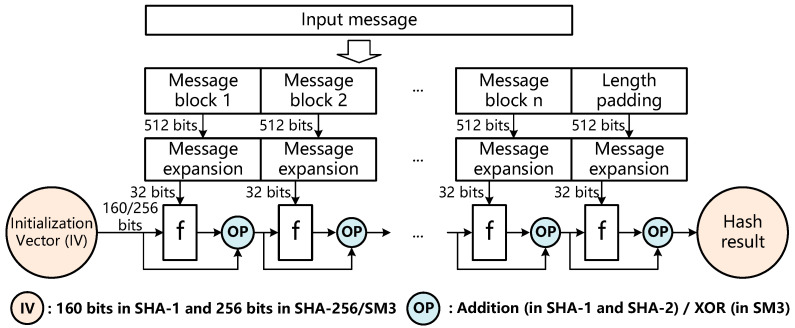
Merkle–Damgård construction of hash functions.

**Figure 4:**
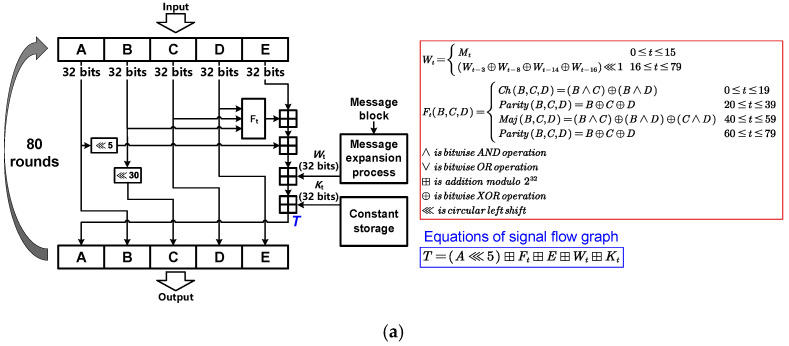

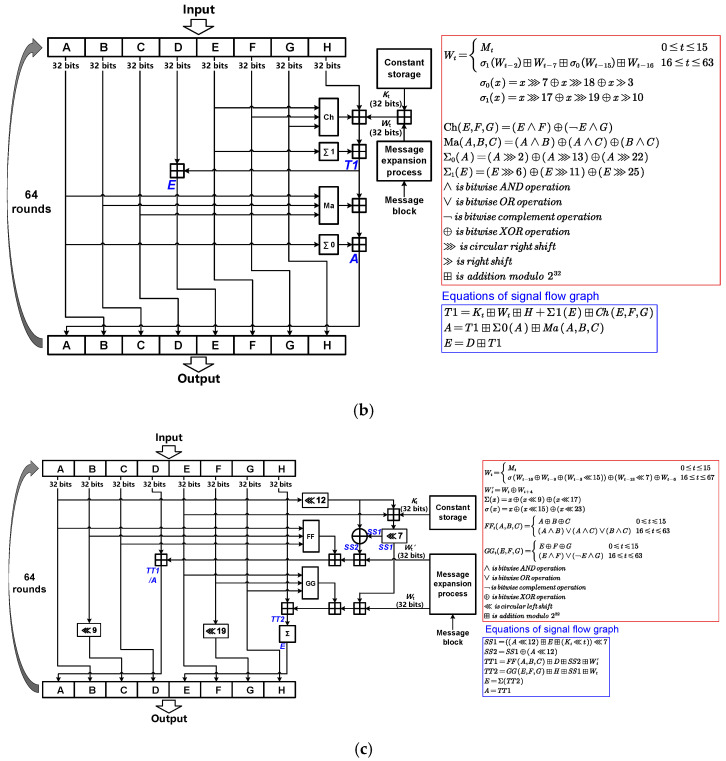
(**a**) The one-way compression function f of SHA-1, (**b**) of SHA-256, (**c**) and of SM3.

Hash functions including SHA-1/SHA-256/SM3 usually adopt the Merkle–Damgård structure [15], as shown in Figure 3. In the Merkle–Damgård diagram, the input message is firstly divided into several blocks of fixed size (512 bits in SHA-1/SHA-256/SM3) and then padded with bits representing the entire message length. The message blocks are processed with a series of cascaded one-way compression functions denoted by f, which transforms two inputs to an output with the same length as the initialization vector (IV), i.e., a fixed value for a specific hash function. For each one-way compression function f, the output should be operated with the input of the same function f by addition in SHA-1/SHA-256 or XOR in SM3, and the result of the operation is taken as the input of the next subsequent compression function f. The one-way compression function f of SHA-1/SHA-256/SM3 is shown in Figure 4. To complete a one-way compression function, the SHA-1, SHA-256 and SM3 need to perform 80, 64 and 64 rounds of operations, respectively. The final output message digest is obtained after all the message blocks with length padding have been compressed by the one-way compression functions.

Figure 4a shows the operation flow in the one-way compression function of SHA-1, with a 160-bit IV and a 160-bit output message digest [5]. For SHA-1, the 160-bit IV is first divided into 5 × 32-bit values and cached by five buffers (A, B, C, D, E). The 512-bit message block is expanded into 80 × 32-bit *K_t_* corresponding to 80 round operations in each one-way compression function of the SHA-1. The 80 round operations are divided into four groups of 20 rounds and each group comprises the same circular left shift operations, the same addition modulo operations and different bitwise logical operations (F_t_). The predefined values of *K_t_* are also different in four different groups of round operations. Each round performs the calculation shown in Figure 4a and updates the five buffers (A, B, C, D, E).

Figure 4b shows the operation flow in the one-way compression function of SHA-256, with a 256-bit IV and a 256-bit output message digest [5]. Different from the SHA-1, the 256-bit IV is first divided into 8 × 32-bit values and cached by eight buffers (A, B, C, D, E, F, G, H). The 512-bit message block is expanded into 64 × 32-bit *W_t_* corresponding to the 64 round operations in each one-way compression function of the SHA-256. Each round operation comprises additions and logical operations, which is more complex than the SHA-1. It is to be noted that the predefined values of *K_t_* are different in each round operation, which is also more complicated than the SHA-1. Each round performs the calculation shown in Figure 4b and updates the eight buffers (A, B, C, D, E, F, G, H).

Figure 4c shows the operation flow in the one-way compression function of SM3, with a 256-bit IV and a 256-bit output message digest [6]. The division of 256-bit IV is the same as SHA-256. The 512-bit message block is expanded into 64 × 32-bit *W_t_* corresponding to the 64 round operations in each one-way compression function of the SM4. The logic operations of message expansion in the SM4 algorithm are different from those in the SHA-256. There are 64 round operations divided into two groups, one for the first 16 rounds and one for the last 48 rounds, respectively. Each group comprises the same circular left shifts, the same addition modulo operations and different bitwise logical operations (FF_t_, GG_t_). Similar to the SHA-256, each round performs the calculation and updates the eight buffers (A, B, C, D, E, F, G, H).

Table 4 summarizes the key operators of round operations in the three major hash functions. The key operators in SHA-1 and SM3 are the same, while the key operators in the SHA-256 round operation are partially different. It is worth noting that all three hash functions use the same Merkle–Damgård construction, which means that the preprocessing (message padding) process of the three hash algorithms is the same and that the corresponding hardware module can be reused. For efficient reconfigurable hardware design of the three hash algorithms, the reuse of modulo addition operator is the key due to its most frequent usage and the most complex operation. Like block ciphers, the reuse of iteration register files and message expansion register files is also the key to reducing hardware overhead and to building a unified data path.

From the above analysis, the key idea of the reconfigurable hardware design of hash functions is to reuse a preprocessing (message padding) module, build a unified compressed data path by reusing register files and modulo addition operators and develop a reconfigurable message expansion module by reusing register files.

#### 2.2.3. Algorithm Comparison between Block Ciphers and Hash Functions

From the aforementioned algorithm analysis and discussion of block ciphers and hash functions, we can summarize three major differences between the two distinctive cryptographic primitives:

- The principle of the block ciphers and hash functions is fundamentally different. From the processing perspective. Block ciphers work in two ways that are reversible, i.e., data encryption and decryption, while hash functions are irreversible that convert original data into message digest. From the application perspective, block ciphers are used to secure the data from the reach of third parties, while hash functions help protect the integrity of the information.
- The structure of round operations in the two cryptographic primitives is different. The round operation structure of the block cipher is based on Feistel or SP, while the round operation structure of hash functions is Merkle–Damgård based on one-way compression. The different structures of the two cryptographic primitives make the hardware data path quite different, which raises a great challenge to the development of a unified data path for efficient reconfigurable hardware design.
- The basic operators in the two cryptographic primitives are different. The key operator of block ciphers is an S-Box performing data substitution, while the key operators of hash functions are logical functions (i.e., F_t_, Ch, Ma, FF_t_, GG_t_). The different basic operators actually impede the reconfigurable design of the two kinds of primitives.

From the hardware design perspective, the three aforementioned major differences make it inefficient to map the two primitives into a unified hardware accelerator. Specifically, a unified hardware accelerator implementing the two different kinds of primitives results in many redundant interconnections, including a large number of multiplexers (MUXs), routers and complex finite state machine logic, or even compilers to realize very complex control. Consequently, the approach of using unified hardware to implement two different kinds of primitives has high hardware complexity, large hardware overhead, and high power consumption.

Furthermore, as mentioned previously, considering many application scenarios of different standards and protocols, it is necessary to perform decryption and hash authentication simultaneously when receiving the ciphertext and message digest at the same time. The approach of using unified hardware requires an operation manner of time division multiplexing, i.e., reconfiguring the hardware into one of either the hash authentication and block decryption mode after the other mode completes its processing task. In contrast, two separate reconfigurable hardware units for these two kinds of primitives can perform decryption and hash authentication at the same time, which improves computing speed and efficiency. Therefore, in this study, two separate reconfigurable cryptographic units are designed to implement block ciphers and hash functions, respectively.

## 3. Hardware Architecture of Proposed Reconfigurable Cryptographic Accelerator

**Figure 5:**
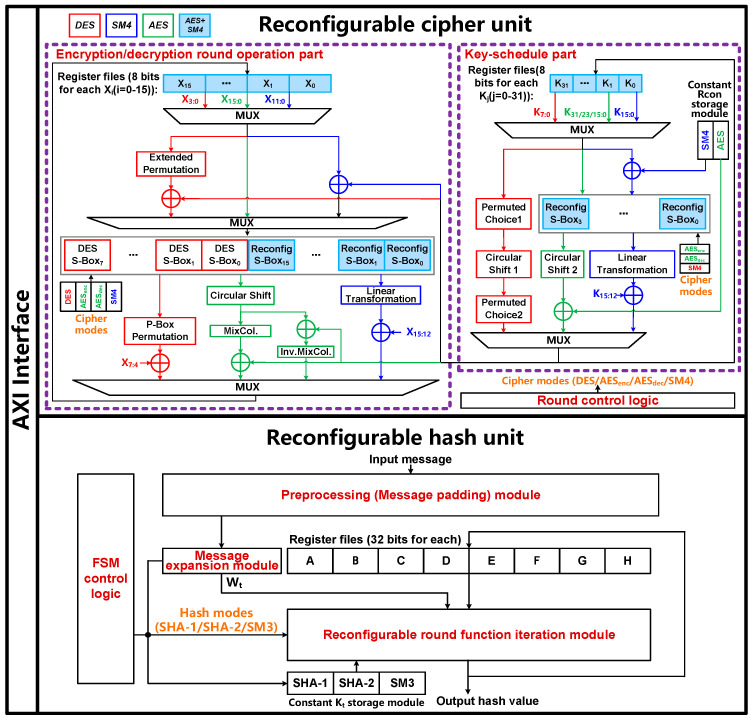
The proposed reconfigurable cryptographic accelerator with reconfigurable cipher unit and reconfigurable hash unit integrated with a unified AXI interface.

Based on the aforementioned analysis of DES/AES/SM4 and SHA-1/SHA-256/SM3 algorithms and the characteristics of operators, a hardware architecture of reconfigurable cryptographic accelerator is proposed, which is suitable for resource-limited and energy-constrained IoT devices including small mobile intelligent robots, wearable medical devices and miniature unmanned aerial vehicles. Specifically, the proposed reconfigurable cryptographic accelerator can be integrated as a dedicated cryptographic engine into System-on-Chip (SoC) chips for robot mapping, positioning and navigation, wearable medical monitoring devices and miniature unmanned aerial vehicles. The accelerator consists of a reconfigurable cipher unit and a reconfigurable hash unit, which are integrated with a unified advanced extensible interface (AXI) as shown in Figure 5.

### 3.1. Reconfigurable Cipher Unit

Figure 5 presents the proposed reconfigurable cipher unit that can perform the encryption and decryption operations of three kinds of block ciphers including DES, AES-128/AES-192/AES-256 and SM4 algorithms. The unit mainly includes the encryption/decryption round operation part, key-schedule part and round control logic, which mainly configures different block ciphers by counting different rounds using a counter to control the reconfigurable cipher unit.

For the encryption/decryption round operation part, the data iteration register files (*X*_0_, *X*_1_, ···, *X*_15_) in light blue color are reused for the DES, AES and SM4 algorithms, and the reconfigurable S-Boxes in light blue color are reused for AES and SM4 algorithms. The three operation modes of this part are described as following:

- In the DES encryption/decryption mode using the logical operators highlighted by red boxes, eight data iteration register files (*X*_0_–*X*_7_) are used to store the 64-bit plaintext. The data path is configured to use the extended permutation module, 48-bit XOR logic, eight S-Boxes, P-Box permutation module and the final 32-bit XOR logic, which are all dedicated to DES due to the distinct difference of operators between DES and AES/SM4.
- In the AES encryption mode using the logical operators highlighted by green boxes, sixteen data iteration register files (*X*_0_–*X*_15_) are used to store the 128-bit plaintext. The data path is configured to use the sixteen reconfigurable S-Boxes, circular shift module, MixColum module and the final 128-bit XOR logic to realize SubBytes, ShiftRows, MixColumns and AddRoundKey, respectively. In AES decryption mode, there are two differences from the AES encryption mode. First, the mode of the sixteen reconfigurable S-Boxes and circular shift module are changed to the AES decryption mode. Second, the MixColum module is replaced by the InvMixColum module according to the AES standard. Note that the modules, including circular shift, MixColum and InvMixColum, are dedicated to the AES mode as there are no such operations in the other two block ciphers.
- In the SM4 encryption/decryption mode using the logical operators highlighted by blue boxes, and as with the AES, sixteen data iteration register files (*X*_0_–*X*_15_) in light blue color are reused to store the 128-bit plaintext. The data path is configured to use the 32-bit XOR logic, four reused reconfigurable S-Boxes, linear transformation module and the final 32-bit XOR logic. Note that the linear transformation module is dedicated to the SM4 mode.

For the key-schedule part, the round key register files (*K*_0_, *K*_1_, ···, *K*_31_) are reused for the DES, AES and SM4 algorithms, and reconfigurable S-Boxes are also reused for AES and SM4 algorithms. The mode analysis is almost the same as the encryption/decryption round operation part shown in Figure 5. It is worth noting that there are 16/24/32 round key register files used for AES-128/192/256 algorithm and four reconfigurable S-Boxes reused for AES-128/192/256 and SM4.

**Figure 6:**
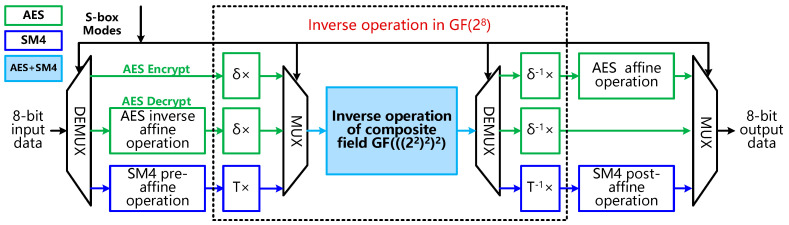
The reconfigurable S-Box module for AES and SM4.

For the design of S-Box, a unified on-the-fly reconfigurable S-Box based on Galois fields (i.e., GF(2^8^)) is designed for the encryption and decryption of AES and SM4 to address the problem of large hardware overhead and non-reusability of S-Box based on LUT implementation. The reconfigurable S-Box module for AES and SM4 is shown in Figure 6. There are 8-bit input data and 8-bit output data for the three operation modes including the AES encryption mode, AES decryption mode and SM4 mode. Among the GF(2^8^) operations, only the inverse operation of composite field GF(((2^2^)^2^)^2^) module in light blue color can be reused for the three modes.

The main idea of designing the reconfigurable S-Box is to reuse the major operation, i.e., the multiplicative inverse operation of AES and SM4 from the analysis of the operations of the S-Boxes in GF(2^8^). We have found that it is necessary to transform the multiplicative inverse operation from GF(2^8^) to the GF(((2^2^)^2^)^2^) composite field because the hardware complexity of the operation in GF(2^8^) is too high. The transformation can be done by the calculation of the isomorphic mapping matrix. Finally, the multiplicative inverse operation in the GF(2^8^) field can be realized by bit-wise logic operations in GF(2^2^) and, therefore, the corresponding efficient hardware architecture of the reconfigurable S-Box can be obtained. The hardware implementation details are described as below.

The S-Box operation of AES encryption includes multiplicative inverse operation in the GF(2^8^) field and affine operation, while the S-Box operation of AES decryption includes inverse affine operation and multiplicative inverse operation in the GF(2^8^) field. The S-Box operation of SM4 encryption/decryption includes pre-affine operation, multiplicative inverse operation in the GF(2^8^) field and post-affine operation. The corresponding operation in the GF(2^8^) field is shown in Equations (1) and (2) [16].

(1) $$Z=M{\left(X\right)}^{-1}+V$$

(2) $$Y=A{\left(AX+C\right)}^{-1}+C$$

where *X* is the input of the S-Box, *Z* and *Y* respectively represent the output of the AES and SM4 S-Box, *M* and *V* represent the affine matrix of the AES S-Box and the constants in the affine operation, respectively, and *A* and *C* represent the affine matrix of the SM4 S-Box and the constants in the affine operation, respectively. The irreducible polynomial (f(*x*)_AES_ and f(*x*)_SM4_), corresponding to the multiplicative inverse operation (*X*)^−1^ and (*AX* + *C*)^−1^ in the finite field GF(2^8^) of AES and SM4 are shown in Equations (3) and (4), respectively.

(3) $$f{\left(x\right)}_{AES}=x^{8}+x^{4}+x^{3}+x^{1}+1$$

(4) $$f{\left(x\right)}_{SM4}=x^{8}+x^{7}+x^{6}+x^{5}+x^{4}+x^{2}+1\;\;$$

From Equations (1) and (2), it can be observed that the multiplicative inverse operation (*X*)^−1^ and (*AX* + *C*)^−1^ in the finite field GF(2^8^) are the main components and similar operations of the two S-Boxes, which can be designed with the reconfigurable method to reduce the hardware overhead. However, the implementation of multiplicative inverse operation in the GF(2^8^) field requires the extended Euclid algorithm, which is computationally intensive and not suitable for hardware implementation [17,18]. According to the properties of the finite field, the operation in GF(2^8^) can be converted to the composite field GF(((2^2^)^2^)^2^) through isomorphic transformation, so as to reduce the amount of computation. The isomorphic mapping from the finite field GF(2^8^) to the composite field GF(((2^2^)^2^)^2^) is represented by an 8 × 8 matrix (matrix *δ* for AES and matrix *T* for SM4) [17]. The 8 × 8 isomorphic mapping matrix *δ* and *T* for AES and SM4 used in this work is shown as follows:

(5) $$\delta =[\begin{array}{cccccccc} 1 & 0 & 0 & 1 & 1 & 0 & 0 & 0\\ 1 & 1 & 1 & 1 & 0 & 0 & 1 & 1\\ 1 & 1 & 1 & 1 & 0 & 0 & 1 & 0\\ 0 & 1 & 0 & 0 & 1 & 0 & 0 & 0\\ 0 & 0 & 0 & 0 & 1 & 0 & 0 & 1\\ 1 & 0 & 0 & 0 & 0 & 0 & 0 & 1\\ 1 & 0 & 1 & 0 & 1 & 0 & 0 & 1\\ 1 & 1 & 1 & 1 & 1 & 1 & 1 & 1 \end{array}],\;\;\;\;T=[\begin{array}{cccccccc} 0 & 0 & 1 & 0 & 0 & 0 & 0 & 1\\ 1 & 1 & 0 & 1 & 0 & 0 & 1 & 1\\ 1 & 0 & 0 & 0 & 0 & 0 & 0 & 1\\ 0 & 1 & 0 & 0 & 1 & 0 & 1 & 0\\ 1 & 0 & 0 & 0 & 1 & 0 & 1 & 0\\ 1 & 0 & 1 & 1 & 1 & 0 & 0 & 1\\ 1 & 0 & 1 & 1 & 0 & 0 & 0 & 0\\ 1 & 1 & 1 & 1 & 1 & 1 & 1 & 1 \end{array}]$$

Assume the inverse of *g* = (*a*_1_*Y*^16^ + *a*_0_*Y*) is *h* = (*d*_1_*Y*^16^ + *d*_0_*Y*), *a*_1_*, a*_0_*, d*_1_*, d*_0_ ∈ GF(2^4^), [*Y*^16^, *Y*] is a set of regular bases in the GF(2^4^) field, and is two roots of irreducible polynomial, i.e., r(*y*) = *y*^2^ + *y* + *η* = 0 (*η* is a constant and is specified for each algorithm). Then the *h* can be calculated as follows:

(6) $$h=\left(d_{1}Y^{16}+d_{0}Y\right)=(\theta^{-1}a_{0})Y^{16}+(\theta^{-1}a_{1})Y,\;\;\theta =\left(a_{1}a_{0}+\left(a_{1}{}^{2}+a_{0}{}^{2}\right)\eta \right)$$

in which way the multiplicative inverse operation in GF(2^8^) can be transformed into operations in GF(2^4^) including multiplication, inverse operation and other operations. Similarly, the inverse of *a* = (*b*_1_*Z*^4^ + *b*_0_*Z*), i.e., *i* = (*e*_1_*Z*^4^ + *e*_0_*Z*), *b*_1_*, b*_0_*, e*_1_*, e*_0_ ∈ GF(2^2^), [*Z*^4^, *Z*] is a set of regular bases in the GF(2^2^) field, and is two roots of irreducible polynomial, i.e., *t(z) = z^2^ + z + ρ* = 0 (*ρ* is a constant and is specified for each algorithm). Then *i* can be calculated as:

(7) $$i=\left(e_{1}Z^{4}+e_{0}Z\right)=(\tau^{-1}b_{0})Z^{4}+(\tau^{-1}b_{1})Z,\;\;\tau =\left(b_{1}b_{0}+\left(b_{1}{}^{2}+b_{0}{}^{2}\right)\rho \right)$$

**Figure 7:**
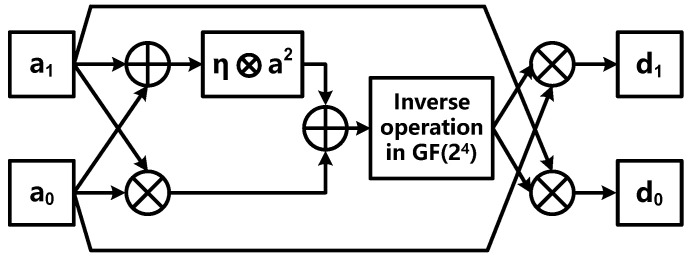
Multiplicative inverse operation of GF(2^8^) based on regular basis.

**Figure 8:**
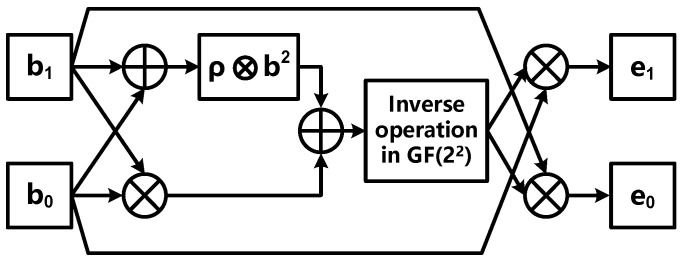
Multiplicative inverse operation of GF(2^4^) based on regular basis.

The operation structure diagram corresponding to Equations (6) and (7) is shown in Figure 7 and Figure 8, respectively.

In GF(2^2^), the multiplicative inverse operation is the same as the square operation. When regular basis is used, the multiplicative inverse operation can be further calculated as:

(8) $${\left(c_{1}W^{2}+c_{0}W\right)}^{-1}={\left(c_{1}W^{2}+c_{0}W\right)}^{2}=\left(c_{0}W^{2}+c_{1}W\right)$$

in which way the multiplicative inverse operation in the GF(2^2^) field can be easily implemented in bit-wise logic operations as mentioned previously.

Table 5 shows the hardware overhead of S-Boxes based on LUT and GF in a 65-nm ASIC implementation. It can be found that the S-Box based on GF(((2^2^)^2^)^2^) field implementation can save hardware overhead by more than 30% against the LUT implementation.

Table 6 shows the comparison of hardware overhead between the reconfigurable cipher unit and the separate hardware implementation of the DES/AES/SM4 in 65 nm, where the gate count is reduced by 33.65%. There are two main reasons for the reduction of hardware overhead. First, the reuse of iteration register files in the unified encryption/decryption and key-schedule data paths allows the number of iteration register files to be reduced by more than 50%. Second, by designing the reconfigurable S-Box based on the composite field GF(((2^2^)^2^)^2^), the gate count of the S-Box can be reduced by about 50% as compared to the conventional LUT implementation method. At the same time, the reuse of S-Boxes by AES and SM4 further achieves a reduction of eight S-Boxes as compared to the separate implementation scheme.

### 3.2. Reconfigurable Hash Unit

As shown in Figure 5, the reconfigurable hash unit can perform three kinds of hash functions including SHA-1, SHA-256 and SM3, which mainly comprises six sub-blocks:

- The preprocessing module mainly performs the original message padding to assure that the input data block is a multiple of 512 bits.
- The message expansion module expands each 512-bit input data block into a predefined round number of words, i.e., round number × *W_t_*, which is 80 × 32 bits in SHA-1 and 64 × 32 bits in SHA-256/SM3.
- The reconfigurable round function iteration module with a unified compressed data path performs 80/64/64 round operations to achieve the one-way compression function f of SHA-1/SHA-256/SM3 for one 512-bit input data block. As a result, the final hash value is obtained after all the 512-bit data blocks have been computed.
- The constant *K_t_* storage module stores the constant *K_t_* used for each round operation.
- The iteration register files (A, B, ···, H) store the initial *IV* in the first round operation and intermediate value obtained from each round operation.
- The finite state machine (FSM) control logic controls the reconfigurable hash unit mainly by generating control signals from counters to control MUXes for realizing reconfigurable computation of the three algorithms.

As mentioned before, the key idea of the reconfigurable hardware design of hash functions is to reuse the preprocessing module, develop the reconfigurable message expansion module by reusing register files, and build a reconfigurable round function iteration module with a unified compressed data path by reusing register files and modulo addition operators.

First, the preprocessing module can directly be reused by SHA-1, SHA-256 and SM3 because the preprocessing procedure is the same as for transforming the input n-bit message into a multiple of 512-bit data blocks by padding for the three hash functions. The padding procedure performs padding on the original n-bit input message by adding one bit of “1”, k bits of “0”, and 64 bits of the original message size, by following the equation of (n + 1 + k = 448 mod 512) to find the minimum k value.

**Figure 9:**
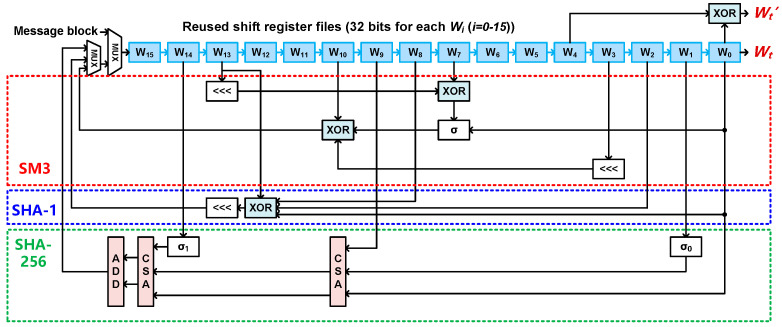
Hardware architecture of message expansion module in the reconfigurable hash unit.

Second, the hardware architecture of the reconfigurable message expansion module is designed to reuse the shift register files (i.e., *W*_0_, *W*_1_, ···, *W*_15_) that are used to store the initial input message of the 512-bit data block from the preprocessing module, and shift the expanded messages during round operations, as shown in Figure 9. For the SM3 mode, the data in the *W*_13_, *W*_10_, *W*_7_, *W*_3_ and *W*_0_ are taken out at each clock cycle to perform circular shift and XOR operation. The σ operator is described by a function containing circular shift and XOR operations in Figure 4c. The output *W_t_* is described by the following Equations (9) and (10):

(9) $$W_{t}=\sigma (W_{t-16}⊕W_{t-9}⊕(W_{t-3}<<<15))⊕(W_{t-13}<<<7)⊕W_{t-6}$$

(10) $$W_{t}{}^{\prime }=W_{t}⊕W_{t+4}$$

For the SHA-1 mode, the data in the *W*_13_, *W*_8_, *W*_2_ and *W*_0_ are taken out at each clock cycle to perform circular shift and XOR operation as described by the following Equation (11):

(11) $$W_{t}=(W_{t-3}⊕W_{t-8}⊕W_{t-14}⊕W_{t-16})<<<1$$

Because the input number of XOR in SHA-1 is different from that of SM3, the XOR operator is not reused for the two operation modes. For the SHA-256 mode, the data in the *W*_14_, *W*_9_, *W*_1_ and *W*_0_ are taken out at each clock cycle to perform the circular shift and XOR operation contained in the functions of σ_0_ and σ_1_. Different from the first two modes of SM3 and SHA-1, the SHA-256 mode has the addition modulo 2^32^ operation as shown in Figure 4b and Equation (12):

(12) $$W_{t}=\sigma_{1}(W_{t-2})+W_{t-7}+\sigma_{0}(W_{t-15})+W_{t-16}$$

Therefore, it is difficult to reuse the operators for message expansion in the three modes for a reconfigurable design. For the three modes, the only shared hardware resources are shift registers, i.e., *W*_15_ to *W*_0_. The expanded value *W_t_* is processed by the logic operations for 80 (in SHA-1) or 64 rounds (in SHA-256 and SM3). For each round, the logic expansion result of *W_t_* is feedback to the input of the register file *W*_15_.

**Figure 10:**
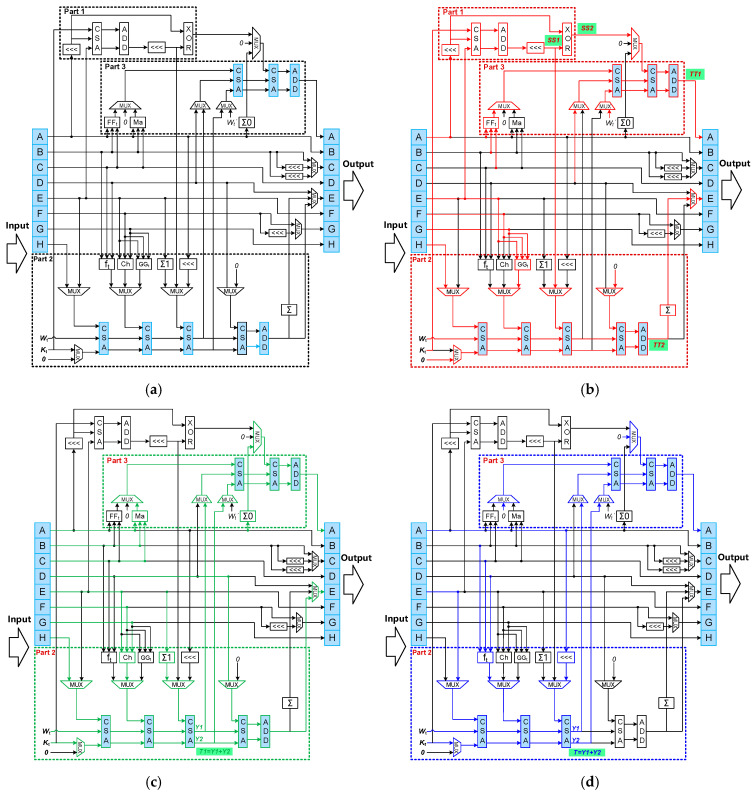
(**a**) Hardware architecture of the proposed reconfigurable round function iteration module in the reconfigurable hash unit, and its three operation modes for (**b**) SM3, (**c**) SHA-256 and (**d**) SHA-1, respectively.

Third, the reconfigurable round function iteration module is designed to build a unified compression data path by reusing register files and modulo addition (sum and modulo 2^32^) operators in light blue color as shown in Figure 10a. Based on the observation that operations in the one-way compression of SM3 contains most operations in both SHA-1 and SHA-256, the main idea is to first design the SM3 data path consisting of part 1, part 2 and part 3 as shown in Figure 10a, and then add a small amount of arithmetic logic and MUXes appropriately to achieve a reconfigurable design supporting the other two hash functions. The module performs multiple rounds of compression operation from the initialization vector (IV) with the expanded value *W_t_* and the constant *K_t_* to calculate the final hash result. The three operation modes of the reconfigurable design are described as following:

- For the SM3 mode, the hardware data path is implemented by direct mapping from the signal flow data path shown in Figure 4d. Specifically, part 1 calculates the intermediate value *SS1* and *SS2* by using constant *K_t_* and values in input register files A and E. At the same time, part 2 calculates the intermediate value *TT2* by using the expanded value *W_t_*, the *SS1* from part 1, and values in input register files E, F, G and H. Part 3 calculates the value *TT1* by using *SS2* from part 1, the expanded value *W_t_*’ and values in input register files A, B, C and D.
- For the SHA-256 mode, three carry save adders (CSAs) in the light blue color are reused to calculate the intermediate value *T1* in part 2. Another CSA and one ADD in light blue color are reused to calculate the value in output register file E in part 2. Two extra CSAs and one ADD in light blue color are also reused to calculate the value in output register file A in part 3. In summary, there are six CSAs and two ADDs reused in the SHA-256 mode. The other logic operators are designed to be dedicated to SHA-256 as they are difficult to be reused for reconfigurable design.
- For the SHA-1 mode, similar to the SHA-256 mode, three CSAs in light blue color are reused to calculate the intermediate value *T* in part 2. Two extra CSAs and one ADD in light blue color are reused to calculate the value in output register file A in part 3. In summary, there are five CSAs and one ADD reused in the SHA-1 mode. The other logic operators are also designed to be dedicated to SHA-1 as they are difficult to be reused for reconfigurable design.

Table 7 shows the comparison of hardware overhead between the reconfigurable hash unit and the separate hardware implementation of the SHA-1/SHA-256/SM3 in 65 nm, where the gate count reduces by 56.18%. There are three main reasons for the reduction of hardware overhead. First, only one preprocessing module is used in the reconfigurable hash unit, while three preprocessing modules are required in the separate implementation scheme. Second, in the message expansion module, sixteen 32-bit register files are reused. Third, in the unified one-way compression data path, registers and addition modulo operators are reused to further reduce the hardware overhead.

## 4. Implementation Results and Discussions

This section presents the results of the proposed reconfigurable cryptographic accelerator based on both Xilinx Virtex UltraScale + Field programmable gate array (FPGA) and 65-nm application-specific integrated circuit (ASIC) implementations. The hardware overhead, speed performance and energy efficiency are presented and discussed comprehensively. The comparison with the existing reconfigurable designs is also performed and discussed.

### 4.1. FPGA Implementation and Evaluation

The separate hardware implementation and reconfigurable implementation of three block ciphers and hash functions are realized based on a Xilinx Virtex UltraScale+ FPGA device. Table 8 shows the hardware overhead of separate hardware implementation and reconfigurable implementation.

For the implementation of three block ciphers, although the LUTs of the reconfigurable implementation increase by 15.0% compared with the separate hardware implementation, the registers, F7 MUXes and F8 MUXes, of the reconfigurable implementation reduce by 17.2%, 90.2% and 100%, respectively, as compared to the separate implementation. There are two main reasons for the reduction of hardware overhead. First, the number of iteration register files are reduced by more than 50% through the reuse of register files. Second, the hardware overhead of the S-Box based on the composite field GF(((2^2^)^2^)^2^) can be reduced by about 30% as compared to the LUT implementation. At the same time, the reuse of S-Boxes by AES and SM4 further achieves a reduction of eight S-Boxes. For the implementation of three hash functions, the LUTs and registers of the reconfigurable implementation reduce by 52.2% and 66.4%, respectively. The implementation results based on FPGA are consistent with the results of ASIC implementation shown in Table 6 and Table 7, both of which show that reconfigurable hardware design has achieved more than 30% and 50% reduction in hardware resource overhead for block ciphers and hash functions, respectively. There are three main reasons for the reduction of hardware overhead. First, only one preprocessing module is used in the reconfigurable hash unit. Second, in the message expansion module, sixteen 32-bit register files are reused. Third, in the unified one-way compression data path, registers and addition modulo operators are reused to further reduce the hardware overhead.

### 4.2. ASIC Implementation and Discussion

**Figure 11:**
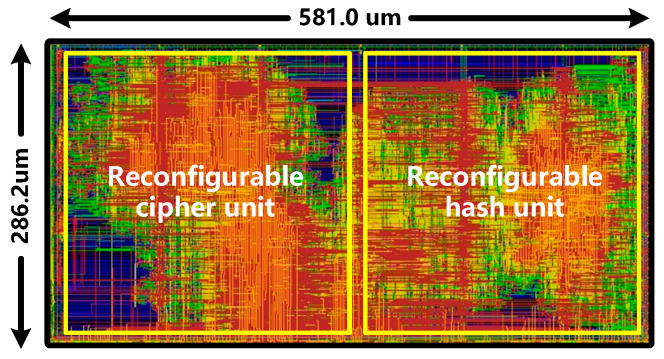
ASIC layout of the proposed reconfigurable cryptographic accelerator core in 65-nm CMOS process.

The proposed reconfigurable cryptographic accelerator core has been implemented into a 65-nm complementary metal oxide semiconductor (CMOS) process technology by a digital ASIC design flow based on Cadence Innovus Place and Route design tool. Figure 11 shows the ASIC layout with a core size of 286.2 μm × 581 μm, where the left part is the reconfigurable cipher unit and the right part is the reconfigurable hash unit.

Table 9 shows the overall comparison between the proposed reconfigurable cryptographic accelerator and the state-of-the-art designs based on ASIC implementation. The benchmark table uses AES-128 algorithm for a fair comparison, which has been widely adopted in many studies [10,11,12].

As shown in Table 9, the proposed reconfigurable cryptographic accelerator achieves the minimum area, the lowest power consumption, and the highest energy efficiency and area efficiency—mainly due to the different implementation methods of various cryptographic algorithms (DES/AES/SM4 and SHA-1/SHA-256/SM3). The implementation method in [10,11,12] is designing a unified reconfigurable engine based on the coarse-grained reconfigurable array (CGRA), which can realize a more flexible reconfigurable architecture supporting various cryptographic algorithms including block ciphers, stream ciphers and hash functions, etc. Specifically, in the three designs, memories are used to store configuration information, FIFOs are used to buffer intermediate data, and reconfigurable processing element arrays including a large number of reconfigurable cells, multiplexers and routers are used to realize a flexible reconfigurable architecture. The works in [10,11,12] have realized a more flexible reconfiguration to support much more cryptographic algorithms, while sacrificing area and energy efficiency, mainly due to the complex routing, interconnections and control logic as compared to our proposed work. On the oth citations power consumption by about 49% and 18%, respectively. The significant reduction mainly benefits from the proposed design methods. These methods entail the building of a unified cipher data path through the reuse of reconfigurable S-Boxes based on Galois field and data iteration register files in the reconfigurable hardware design of block ciphers. They also entail the reuse of a preprocessing (message padding) module, the building of a unified compressed data path, and the development of a reconfigurable message expansion module in the reconfigurable hardware design of hash functions.

For a case study of energy efficiency improvement for the proposed reconfigurable cryptographic accelerator, a typical lithium coin battery with 37 mAh is selected [19]. For a 3-lead 360-Hz 12-bit electrocardiogram (ECG) recording system, there are about 131 MBytes of raw data for one day, which needs to be processed by AES-128 and SHA-256. The 65-nm ECG SoC in [20] is selected to integrate the cryptographic accelerator. For a smart sensor node, usually 20% of battery power [21] can be allocated by the ECG SoC, which consumes 0.254 J per day when the cryptographic accelerator is not integrated. The 65-nm design in [11] is selected for comparison, which has the highest energy efficiency among the three designs in [10,11,12]. For the AES-128 cipher block processing of the 131 MBytes of raw data, the energy consumption per day is 0.00325 J and 0.01 J, by the design in [11] and our proposed design, respectively. For the SHA-256 hash function, the energy consumption per day is 0.0045 J and 0.735 J by the design in [11] and our design, respectively. From our calculation, when integrating the accelerator in [11] and our proposed cryptographic accelerator, the endurance time of the ECG SoC is estimated to 53.33 days and 203.55 days, respectively. The results show that the ECG SoC integrated with our proposed cryptographic accelerator can significantly increase the endurance time by more than ten times.

For the application scenarios where both decryption and hash authentication are required to operate simultaneously, the latency of the proposed reconfigurable cryptographic accelerator is much lower than the state-of-the-art designs in [10,11,12]. The comparison of processing latency with [11] is performed because only the work in [11] presents the cycles of mode configuration and algorithm acceleration. This comparative study uses the following scenario as an example: the cryptographic SoC receives a 512-bit ciphertext and a 256-bit hash value simultaneously, and, therefore, it needs to use AES-128 and SHA-256 for decryption and hash authentication, respectively. For the design in [11], the reconfigurable cryptographic accelerator takes 367 cycles to be configured in the AES decryption mode, 80 (20 × 4) cycles to perform decryption, 370 cycles to be configured in the SHA-256 mode, and 160 (80 × 2) cycles to perform hash authentication. In total, it takes 977 cycles for the design in [11] to complete the decryption and hash authentication tasks in the aforementioned scenario in practical IoT applications. For our reconfigurable cryptographic accelerator design, it takes only two cycles to configure the reconfigurable cipher unit and reconfigurable hash unit to the AES-128 decryption mode and the SHA-256 mode through the AXI bus, respectively. Subsequently, the reconfigurable cipher unit takes 80 (20 × 4) cycles to perform decryption and the reconfigurable hash unit takes 160 (80 × 2) cycles to perform hash authentication simultaneously. As compared to the existing reconfigurable design for both block cipher and hash function algorithms in a single unified data path, our proposed design is able to complete the acceleration of computing block cipher and hash function algorithms at the same time by much shorter processing latency (i.e., a total of 162 cycles in the above case study), which can significantly reduce the number of processing cycles (i.e., 83% reduction as compared to the state-of-the art design in [11]).

In a summary, the above comparison and evaluation against the state-of-the-art designs has shown that the proposed reconfigurable cryptographic accelerator design have achieved higher area efficiency, better energy efficiency and faster processing speed, which is more suitable for power-constrained and cost-sensitive IoT applications.

## 5. Conclusions

The paper presents the design and analysis of a reconfigurable cryptographic accelerator that consists of a reconfigurable cipher unit and a reconfigurable hash unit with a unified cipher data path and a unified compression data path, respectively. The solution of mapping block ciphers and hash functions to two separate reconfigurable units proposed in this work significantly reduces the number of cycles, by 83% compared with a unified reconfigurable engine solution in the literature, when both block ciphers and hash functions are required to operate at the same time in many IoT application scenarios. The two unified data paths are proposed by reusing the key registers and operators to reduce the hardware overhead. The reconfigurable S-Box module based on GF(((2^2^)^2^)^2^) is designed and reused to further reduce the hardware overhead by more than 30% compared with the conventional LUT solution. Thanks to the hardware architecture with two separate reconfigurable units and the reconfigurable design methods mentioned above, the energy efficiency and area efficiency of the proposed design in 65-nm CMOS technology has achieved 441 Gbps/W and 37.55 Gbps/mm^2^, respectively. The high energy efficiency and area efficiency show that the proposed reconfigurable cryptographic accelerator is very attractive for many IoT devices with limited battery and form factor including intelligent small mobile intelligent robots, wearable medical devices and miniature unmanned aerial vehicles.

Because cryptographic chips are at risk of side channel attacks [22], we will further increase the capability of resisting side channel attacks in the future to implement a more secure cryptographic chip.

## Figures and Tables

**Table 1:** Major algorithms in block ciphers and hash functions.

	Algorithms	Security Protocols	Applicable Countries/Regions	Organization	Typical Application Example
Block Cipher	DES	IPsec/SSL/TLS	USA/CHN/EUR	NIST	Ethernet
	AES	Most communication standards/protocols	USA/CHN/EUR	NIST	WLAN/ZigBee/Bluetooth/5G/LTE/Ethernet
	SM4	IPsec/TLS/DTLS/SSL/WAPI	CHN	SCA	WLAN/Ethernet
Hash function	SHA-1	IPsec/SSL/TLS	USA/CHN/EUR	NIST	RFID/Ethernet
	SHA-256	Most communication standards/protocols	USA/CHN/EUR	NIST	WLAN/Bluetooth/5G/LTE/Ethernet
	SM3	IPsec/SSL/TLS	CHN	SCA	WLAN/Ethernet

NIST: National Institute of Standards and Technology. SCA: State Cryptography Administration.

**Table 2:** Structure types and key operators of round operations in the three major block ciphers.

Block Ciphers	Round Operation Structure Type	Key Operators in Round Operation
DES	Feistel	XOR/S-Box/Permutation
AES	SP	XOR/S-Box/Shift/Column Mix
SM4	Feistel	XOR/S-Box/Shift

**Table 3:** Detailed information on key operators of round operations in the three major block ciphers.

Key Operators	Input/Output Bit-Widths	Number of Operators Used in One Round Operation of Block Cipher		
		DES	AES	SM4
Permutation	64-56 bits	1	-	-
	56-48 bits	1	-	-
	32-48 bits	1	-	-
	32-32 bits	1	-	-
XOR	48 bits	1	-	-
	128 bit	-	1	1
	32 bit	1	4	14
S-Box	6-4 bit S-Box	8	-	-
	8-8 bit S-Box	-	20	8
Shift	28-bit circular left shift	2	-	-
	32-bit circular left shift	-	4	6
Column Mix (Inverse Column Mix)	32-bit (Inverse) column mix	-	4	-

**Table 4:** Detailed information of key operators in the three major hash functions.

Key Operators	Input and Output Bit-Widths	Number of Operators in One Round Operation of Hash Functions		
		SHA-1	SHA-256	SM3
XOR	32 bit	5	11	10
Shift	32-bit circular left shift	3	-	11
	32 bits circular right shift	-	10	-
	32 bits right shift	-	2	-
Modulo addition	32 bits ADD mod 2^32^	4	9	7

**Table 5:** Hardware overhead of S-Boxes based on LUT and GF(((2^2^)^2^)^2^) in 65-nm ASIC implementation.

Algorithm Type	Implementation	Gate Count	Saving
AES	LUT	586	N/A
	GF	335	42.83%
SM4	LUT	368	N/A
	GF	244	33.70%

**Table 6:** Hardware overhead of separate hardware implementation and reconfigurable implementation of the three block ciphers in 65 nm.

Structure Type	Algorithm Type	Gate Count	Saving
Separate	DES-64	1956	N/A
	AES-128/192/256	19,518	N/A
	SM4-128	5635	N/A
Total	DES+AES+SM4	27,109	N/A
Reconfigurable	DES/AES/SM4	17,986	33.65%

**Table 7:** Hardware overhead of separate hardware implementation and reconfigurable implementation of the three hash functions in 65 nm.

Structure Type	Algorithm Type	Gate Count	Saving
Separate	SHA-1	16,055	N/A
	SHA-256	18,627	N/A
	SM3	17,921	N/A
Total	SHA-1+SHA-256+SM3	52,603	N/A
Reconfigurable	SHA-1/SHA-256/SM3	23,050	56.18%

**Table 8:** Hardware overhead of the separate hardware implementation and the reconfigurable implementation of the three block ciphers and hash functions based on FPGA.

Structure Type	Algorithm Type	LUTs	Registers	F7 MUXes	F8 MUXes
Separate	DES-64	342	74	96	0
	AES-128/192/256	3756	685	649	256
	SM4-128	732	266	103	20
Total	DES+AES+SM4	4830	1025	848	276
Reconfigurable	DES/AES/SM4	5555	849	83	0
Separate	SHA-1	1980	2910	0	0
	SHA-256	2549	2922	0	0
	SM3	2043	2939	0	0
Total	SHA-1+SHA-256+SM3	6572	8771	0	0
Reconfigurable	SHA-1/SHA-256/SM3	3140	2951	0	0

**Table 9:** Overall comparison of the proposed reconfigurable cryptographic accelerator and existing designs based on ASIC implementation.

	TCAD’2018 [10]	TCAS-II’2020 [11]	TVLSI’2020 [12]	Ours Proposed Design
Major key algorithms	DES/AES/SM4 ZUC/SNOW/RC4 SHA-256/SM3	DES/AES/SM4 ZUC/SNOW/RC4 SHA-256/SM3	DES/AES/SM4 ZUC/RC4 SHA-256	DES/AES/SM4 SHA-1/SHA-256/SM3
Architecture	Reconfigurable architecture based on one unified data path	Reconfigurable architecture based on one unified data path	Reconfigurable architecture based on one unified data path	Reconfigurable architecture based on two unified data paths for cipher block and hash function algorithms
Technology	65 nm	65 nm	55 nm	65 nm
Area (mm^2^)	7.75	9.91	12.25	0.11
Gate count	1910 K	N/A	N/A	41 K
Memory	97.9	N/A	N/A	0
Frequency (MHz)	400	500	110	323
Power (mW)	620 (155 *)	625 (125 *)	35 (31.81 *)	23 (7.12) *
Throughput (Gbps)	51.2 (12.8 *)	64 (12.8 *)	0.44 (0.4 *)	4.13 (1.28) *
Energy efficiency (Gbps/W)	82.6	102.4	15.71	441
Area efficiency (Gbps/mm^2^)	6.61 (1.65 *)	6.46 (1.29 *)	0.04 (0.03 *)	37.55 (11.64) *
S-Box topology	LUT	LUT	LUT	GF(((2^2^)^2^)^2^)

*: scaled to 100 MHz.
